# Selenium nanoparticles derived from *Proteus mirabilis* YC801 alleviate oxidative stress and inflammatory response to promote nerve repair in rats with spinal cord injury

**DOI:** 10.1093/rb/rbac042

**Published:** 2022-06-23

**Authors:** Xiangyu Liu, Yingji Mao, Shengwei Huang, Weifeng Li, Wei Zhang, Jingzhou An, Yongchao Jin, Jianzhong Guan, Lifang Wu, Pinghui Zhou

**Affiliations:** Department of Orthopedics, First Affiliated Hospital, School of Life Sciences, Bengbu Medical College, Bengbu, Anhui 233004, China; Department of Orthopedics, First Affiliated Hospital, School of Life Sciences, Bengbu Medical College, Bengbu, Anhui 233004, China; Anhui Province Key Laboratory of Tissue Transplantation, Bengbu Medical College, Bengbu, Anhui 233030, China; Institute of Biomedical and Health Science, School of Life and Health Science, Anhui Science and Technology University, Fengyang, Anhui 239000, China; Department of Orthopedics, First Affiliated Hospital, School of Life Sciences, Bengbu Medical College, Bengbu, Anhui 233004, China; Department of Orthopedics, First Affiliated Hospital, School of Life Sciences, Bengbu Medical College, Bengbu, Anhui 233004, China; Department of Orthopedics, First Affiliated Hospital, School of Life Sciences, Bengbu Medical College, Bengbu, Anhui 233004, China; Department of Orthopedics, First Affiliated Hospital, School of Life Sciences, Bengbu Medical College, Bengbu, Anhui 233004, China; Anhui Province Key Laboratory of Tissue Transplantation, Bengbu Medical College, Bengbu, Anhui 233030, China; The Center for Ion Beam Bioengineering and Green Agriculture, Hefei Institutes of Physical Science, Chinese Academy of Sciences, Hefei, Anhui 230031, China; Department of Orthopedics, First Affiliated Hospital, School of Life Sciences, Bengbu Medical College, Bengbu, Anhui 233004, China; Spinal Deformity Clinical Research Center of Anhui Province, Fuyang 236000, China

**Keywords:** biogenic selenium nanoparticle, microglia polarization, nerve renovation, neural stem cells, neuroinflammation, oxidative stress, spinal cord injury

## Abstract

Microbial biotransformation and detoxification of biotoxic selenite into selenium nanoparticles (SeNPs) has emerged as an efficient technique for the utilization of selenium. SeNPs are characterized by high bioavailability and have several therapeutic effects owing to their antioxidant, anti-inflammatory and neuroprotective activities. However, their influence on microenvironment disturbances and neuroprotection after spinal cord injury (SCI) is yet to be elucidated. This study aimed to assess the influence of SeNPs on SCI and explore the underlying protective mechanisms. Overall, the proliferation and differentiation of neural stem cells were facilitated by SeNPs derived from *Proteus mirabilis* YC801 via the Wnt/β-catenin signaling pathway. The SeNPs increased the number of neurons to a greater extent than astrocytes after differentiation and improved nerve regeneration. A therapeutic dose of SeNPs remarkably protected the integrity of the spinal cord to improve the motor function of the hind limbs after SCI and decreased the expression of several inflammatory factors such as tumor necrosis factor-α and interleukin-6 *in vivo* and enhanced the production of M2-type macrophages by regulating their polarization, indicating the suppressed inflammatory response. Besides, SeNPs reversed the SCI-mediated production of reactive oxygen species. In conclusion, SeNPs treatment holds the potential to improve the disturbed microenvironment and promote nerve regeneration, representing a promising therapeutic approach for SCI.

## Graphical Abstract

**Figure:**
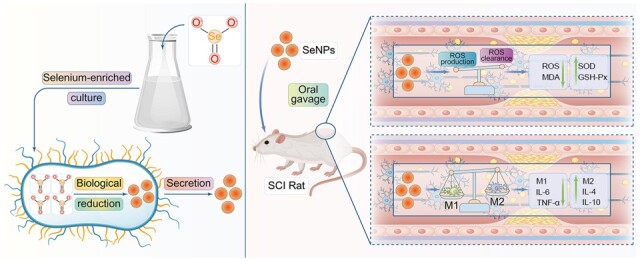

## Introduction

As an essential trace element in the human body, Selenium (Se) showed significant health benefits, including enhancing immune capacity and resisting free radical damage [1]. However, the safe range between the beneficial and toxic doses of Se is narrow [2, 3]. Compared with inorganic selenium, nontoxic elemental selenium (Se^0^) such as selenium nanoparticles (SeNPs) has been found to possess low toxicity, acceptable bioavailability and high efficiency in preventing oxidative damage, thereby attracting worldwide attention for their application as therapeutic agents [4–7]. They are involved in several important metabolic and physiological processes, including antioxidant defense and immune regulation. In addition, nanoparticles with strong catalytic performance can directly penetrate biological tissues and cells, suggesting that SeNPs may have high efficiency in inhibiting inflammation and oxidative stress than inorganic selenium [8]. Due to their unique advantages, SeNPs have attracted great attention in the treatment of central nervous system (CNS) diseases in recent years.

The administration of appropriate SeNPs had a therapeutic effect on Alzheimer’s disease by lowering reactive oxygen species (ROS) and H_2_O_2_ levels [9]. SeNPs could also be used to treat cypermethrin-induced neurotoxicity by resolving the complex immune environment, such as decreasing malondialdehyde (MDA) and tumor necrosis factor-α (TNF-α) levels [10]. In a *Caenorhabditis elegans* model, SeNPs could reverse Huntington’s disease (HD) and enhance survival [11]. These findings suggested that SeNPs are a promising therapeutic tool for neurological diseases. However, the application of biogenic SeNPs to spinal cord injury (SCI) remains to be explored.

SCI is a neurodegenerative disease that often occurs in young and middle-aged adults [12, 13]. The underlying mechanisms of SCI include oxidative stress, inflammatory response, excitotoxicity, apoptosis and microcirculation disturbances [14]. After SCI, inflammation not only has a scavenging effect on necrotic tissue and cells but also leads to the formation of cavities and glial scars, inhibiting the recovery of neurological function. Macrophages are one of the first activated inflammatory cells and play an important role in the inflammatory process [12]. As the predominant effector cells, macrophages enter the injured site and polarize into M1 and M2 type in inflammatory responses [15, 16]. Activated M1-type macrophages are characterized by the production of ROS that indicates a rapid and powerful response to trigger inflammation [17–19]. They release inflammatory cytokines like TNF-α and interleukin (IL)-6 to activate neutrophils and lymphocytes to produce an inflammatory cascade in the injury site [16, 19]. On the other hand, IL-10 and IL-4 receptor antagonists produced by M2-type macrophages attenuate the extent of SCI and thus show a positive effect on the repair and regeneration after SCI [12]. In this process, superoxide dismutase (SOD) is the predominant antioxidant enzyme for eliminating free radicals in cells, while glutathione peroxidase (GSH-Px) eliminates the lipid peroxides and protects the cell membrane integrity [20]. Studies have shown that the timely inhibition of injury-related immunological mechanisms by attenuating oxidative stress and inflammation can minimize neuronal cell apoptosis and promote axon regeneration after the injury and may be a valuable approach to alleviate the neurological impairment associated with SCI [21, 22].

Many attempts have been made to repair SCI using various biomaterials, including combining biological scaffolds and natural drugs to promote tissue regeneration. Biological scaffolds have good cell affinity and biodegradation characteristics, which favors tissue repair. However, their rapid biodegradability also hinders their application in various manufacturing technologies. In contrast, natural drugs, because of their simplicity and controllability, are promising methods for repairing SCI damage cascades by reducing apoptosis and inflammation and improving oxidative stress status [23].

SeNPs derived from bacteria are superior to those from physical or chemical origins due to their low cost, low environmental hazard and high biocompatibility *in vivo* [24, 25]. In a previous study, our research group successfully developed a method to prepare biogenic SeNPs with *Proteus mirabilis* YC801, which could reduce sodium selenite (Na_2_SeO_3_) into SeNPs with detoxification [26]. Bacteria-derived biogenic SeNPs had extremely high biocompatibility and thus have shown promise for the treatment of inflammation- and oxidative stress-related disorders [27]. Hence, in the present study, the biogenic SeNPs were used to explore their potential value in spinal cord repair in SCI rats.

## Materials and methods

### Preparation of biogenic SeNPs

The biogenic SeNPs were prepared referring to the previous study [26]. Briefly, *P.mirabilis* YC801 was cultured in a 500-ml flask with 200 ml YEP medium and 5 mM Na_2_SeO_3_ at 30°C for 36 h. The bacterial cultures were centrifuged at 10 000×g at 4°C for 10 min, and the precipitate was collected and washed twice with 0.9% sodium chloride (NaCl) solution to remove the contamination of Na_2_SeO_3_. Then, the precipitate was resuspended in 20 ml Tris-HCl buffer (50 mM, pH = 8.2) and ultrasonically crushed on ice for 20 min (ultrasonic for 40 s, stop for 40 s). Subsequently, the suspension was centrifuged at 12 000×g for 40 min, followed by centrifugation at 40 000×g for 4°C for 40 min to collect the nanoparticles. Finally, the SeNPs were washed twice with 0.9% NaCl and resuspended in deionized water.

### Characterization of biogenic SeNPs

The following methods were applied to evaluate the location, distribution and characterization of biogenic SeNPs.

#### Transmission electron microscope

After culturing 24 h with 5 mM Na_2_SeO_3_, the fresh bacterial cultures were taken out and centrifuged at 5000×g for 6 min. The isolate cultured in only YEP medium served as a negative control. The pellets were suspended with fresh precooled 2.5% glutaraldehyde in 0.1 M phosphate-buffered saline (PBS, pH 7.4) and fixed overnight at 4°C. Subsequently, the samples were placed on the copper grid and dried at room temperature. The micrograph of the sample was obtained by HT-7700 (Hitachi, Tokyo, Japan) transmission electron microscope (TEM) at 80 kV.

#### Scanning electron microscopy-energy dispersive X-ray

As mentioned in 2.2.1, the fixed samples were washed twice in 0.1 M PBS and treated with an ascending series of ethanol (30%, 50%, 70%, 80%, 90%, and 100%) for gradient dehydration. Then, samples were dried using CO_2_ supercritical drying and coated with a gold layer. The imaging was taken using the S4800 (Hitachi, Tokyo, Japan) scanning electron microscopy (SEM) at 3 kV. Qualitative analysis of elements was analyzed by SEM equipped with an Oxford Instruments energy dispersive X-ray (EDX) analyzer at 12 keV.

For the SEM characterization, the SeNPs suspension was first free-dried and then subjected to the scanning electron microscopy-energy dispersive X-ray analysis.

#### Dynamic light scattering

To investigate the size distribution of SeNPs, the dynamic light scattering (DLS) was performed using Zen3600 Zetasizer Nano-ZS (Malvern, Worcestershire, UK). The purified SeNPs were suspended in distilled water, mixed by ultrasonic treatment and diluted 1:10–1:20 to meet the optical requirements of the instrument. The determination was recorded using the software equipped by Malvern Instruments and repeated three times.

### *In vitro* experiments

#### Neural stem cells isolation and culture

Fetal Sprague–Dawley (SD) rats were provided by the Pengyue Laboratory Animal Breeding Co. Ltd (Shandong, China). All animal protocols were approved by the Animal Care and Use Committee of Bengbu Medical College (Approval Number: 2020238, Bengbu, China). Neural stem cells (NSCs) were obtained from pregnant rats, 8–10 weeks old. After the pregnant rats were sacrificed by cervical dislocation, the fetal rats were removed from the uterus on an aseptic table and soaked in 75% alcohol for 5 min. The head was isolated and then quickly soaked in PBS buffer (pH 7.4). After removing the skull and meninges on a super-clean worktable, the brain was cut into pieces in Dulbecco’s Modified Eagle Medium/Nutrient Mixture F-12 (DMEM/F12, Gibco, Invitrogen, USA) culture medium. Next, the obtained brain solution was centrifuged (110 g) for 5 min. Then the sediment was resuspended in the 4 ml serum-free medium (DMEM/F12, 2% B27 supplement, and 1% penicillin-streptomycin solution) after removing the supernatant. After cell counting, the concentration was adjusted to 1.0 × 10^9^·l^−^^1^, and the cells were inoculated in a 25 cm^2^ culture flask. Next, the flasks were incubated at 37°C in humidified air of 50% containing 5% CO_2_. The medium was changed every 3 days and cells were used in passage 3 [28].

#### NSCs proliferation and differentiation

The Cell Counting Kit-8 (CCK-8; Biosharp, HeFei, China) assay and the immunofluorescence tests were used to assess the effects of biogenic SeNPs on NSCs proliferation and differentiation. For NSCs proliferation, the NSCs were seeded in 96-well plates (8 × 10^3^ cells/well), which were pretreated with a poly-*D*-lysine solution (0.1 mg/ml; BBI Life Sciences, Shanghai, China) and were incubated in 5% CO_2_ at 37°C overnight. After the suspended cells completely adhered to the bottom walls, the NSCs culture medium (DMEM/F12 with 2% B27 supplement and 1% penicillin-streptomycin solution) with an increasing concentration of the SeNPs (0, 5, 10 or 20 μg/ml) was added to the wells. On Days 1, 4 and 7 of culture, CCK-8 reagent was added to each well, and the absorbance at 450 nm was measured after 10 s of oscillation. NSCs cultured without SeNPs served as a control group.

For NSCs differentiation, 8 × 10^3^ cells/well were cultured in 96 well plates with an NSC differentiation medium (DMEM/F12, 2% B27 supplement, 1% fetal bovine serum and 1% penicillin-streptomycin solution) containing different concentrations of SeNPs (0, 5, 10 or 20 μg/ml). After 7 days of culture, the cells were incubated with antibodies against beta-tubulin III (Tuj-1, 1:200; Biosharp, Hefei, China) and glial fibrillary acidic protein (GFAP, 1:200; Biosharp), and then with the corresponding secondary antibody (Cyanine 3, 1:200; Biosharp). To quantify NSCs differentiation, Tuj-1-positive and GFAP-positive neurons and astrocytes were counted using Fluorescence Inverted Microscope (Zeiss, Oberkochen, Germany).

#### Western blotting

To assess the mechanisms of SeNPs induced NSCs proliferation and differentiation, the related proteins in NSCs after treatment were measured by western blotting. Briefly, there were three groups, including the Control group, SeNPs group and SeNPs+Dkk-1 (100 nM) group (Dkk-1, the Wnt pathway inhibitor) [29]. After 7 days cultured, the protein extracted with RIPA (Biosharp, Heifei, China) was separated on sodium dodecyl sulfate polyacrylamide gel electrophoresis (SDS-PAGE) gels and transferred onto polyvinylidene difluoride (PVDF) membranes (Millipore, USA). After blocking with 5% skimmed milk and 0.1% Tween 20 in Tris buffer solution (TBST) for 1 h at room temperature, the membranes were incubated with primary antibodies against Wnt-3 (1 µg/ml, Abcam, Cambridge, UK), glycogen synthase kinase (GSK)-3 (1:1000, Abcam, Cambridge, UK), p-GSK-3 (1:1000, Abcam, Cambridge, UK), β-catenin (1:5000, Abcam, Cambridge, UK), p-β-catenin (1:1000, Abcam, Cambridge, UK), cyclin D (1:100, Abcam, Cambridge, UK), neuro D1 (1:1000, Abcam, Cambridge, UK) and neurogenin (1:5000, Abcam, Cambridge, UK) overnight at 4°C. Then, the membranes were incubated with horseradish peroxidase-conjugated IgG antibody at room temperature for 1 h. The results were normalized using β-actin (1:1000, Abcam, Cambridge, UK) as the internal reference, and quantification of the protein levels was obtained using Image J (National Institutes of Health).

### *In vivo* experiments

#### Animals

Two-month-old female SD rats (190–210 g) were obtained from Pengyue Laboratory (Shandong, China). The rats were bred in standard temperature conditions (23–25°C) with a 12 h light/dark cycle and a 40–60% relative humidity and had free access to food and water.

#### Establishment of rat SCI model

The contusive SCI model was established using an NYU impactor (NYU, New York) with a 10 g rod dropped onto the T9 spinal cord from a height of 12.5 mm [30]. Briefly, the animals were firstly anesthetized with phenobarbital (50 mg/kg). After the T9 lamina was excised on the operating table, the spinal cord was exposed, then the T8 and T11 spinous processes were clamped with the impactor. The rod (1.3 mm in diameter) was used to hit the spinal cord to establish the SCI model.

After the surgery, the rats were bred at a temperature of 25°C and 40–60% humidity. For the SeNPs therapy assay, the rats were randomly separated into four groups (*n* = 10 in each group): the SCI group received by gavage of 1 ml of normal saline solution, and the other three groups were administered by gavage of 1 ml of biogenic SeNPs at a concentration of 0.5, 1.0 or 2.0 mg/kg, respectively. The rats were injected intramuscularly with antibiotics (2.5 mg/kg; Enrofloxacin 10%) for 7 days to prevent infection. The bladder was squeezed twice daily to assist urination until normal excretion was restored.

#### Motor behavior analysis

The behavioral evaluation was performed using the Basso Beattie Bresnahan (BBB) locomotor rating scale, a 21-point scale based on observations of the crawling ability of rats freely moving in an open field. The BBB scores were observed at 0, 1, 3, 7, 14, 21 and 28 days following an injury. During the tests, the rats were moved freely on an open-field surface for 5 min while being observed by two blinded evaluators.

#### Enzyme-linked immunosorbent assay

Inflammatory cytokines released in response to SCI were evaluated 7 days after the surgery. Briefly, serum was collected, and the levels of TNF-α (1317202), IL-6 (1310602), IL-4 (1310402) and IL-10 (1311002) were determined using commercial enzyme-linked immunosorbent assay kits (DAKEWEI, Beijing, China) according to the manufacturer’s instructions.

#### Histological analysis

All tissue samples were removed 7 and 28 days after injury for histological analysis. As previously described [28], the samples were longitudinally cut into serial sections of 9 μm thickness and were stained with hematoxylin and eosin (H&E). Then, the spinal cord sections were observed and photographed under the inverted fluorescence microscope (IX-71, Olympus, Tokyo, Japan).

#### Estimation of oxidative stress activity

The levels of ROS, MDA, SOD and GSH-Px were assessed using commercial kits to estimate internal oxidative stress levels (Jiancheng BI, Nanjing, China). Briefly, blood samples and spinal cord tissues were collected and processed 14 days after surgeries according to the manufacturer’s instructions [31].

#### Immunohistofluorescence staining

Spinal cord tissue sections were blocked and incubated with the following primary antibodies: anti-CD68 (1:100), anti-C–C motif receptor (CCR)7 (1:500), anti-arginase (Arg)-1 (1:1000), anti-Tuj-1 (1 µg/ml), anti-chondroitin sulfate (CS-56) (2.5 µg/ml), anti-laminin (1:50), anti-GFAP (1:1000), anti-tyrosine hydroxylase (TH) (1:100) and anti-neurofilament (NF) (1:500) (Abcam, Cambridge, UK). The following day, the samples were stained with the respective secondary antibodies (Cyanine 3, 1:200; Biosharp, Alexa Fluor^®^ 488, 1:200; Biosharp). Finally, the sections were sealed and images were obtained using a fluorescence microscope (Olympus, Tokyo, Japan).

### Statistics

All data were analyzed using the SPSS software (version 20.0; IBM, Armonk, NY, USA). Data are presented as mean ± standard deviation and were evaluated by one-way analysis of variance. Differences were considered statistically significant at *P*-values <0.05.

## Results

### Localization and characterization of SeNPs produced by *P.mirabilis* YC801

**Figure 1.**
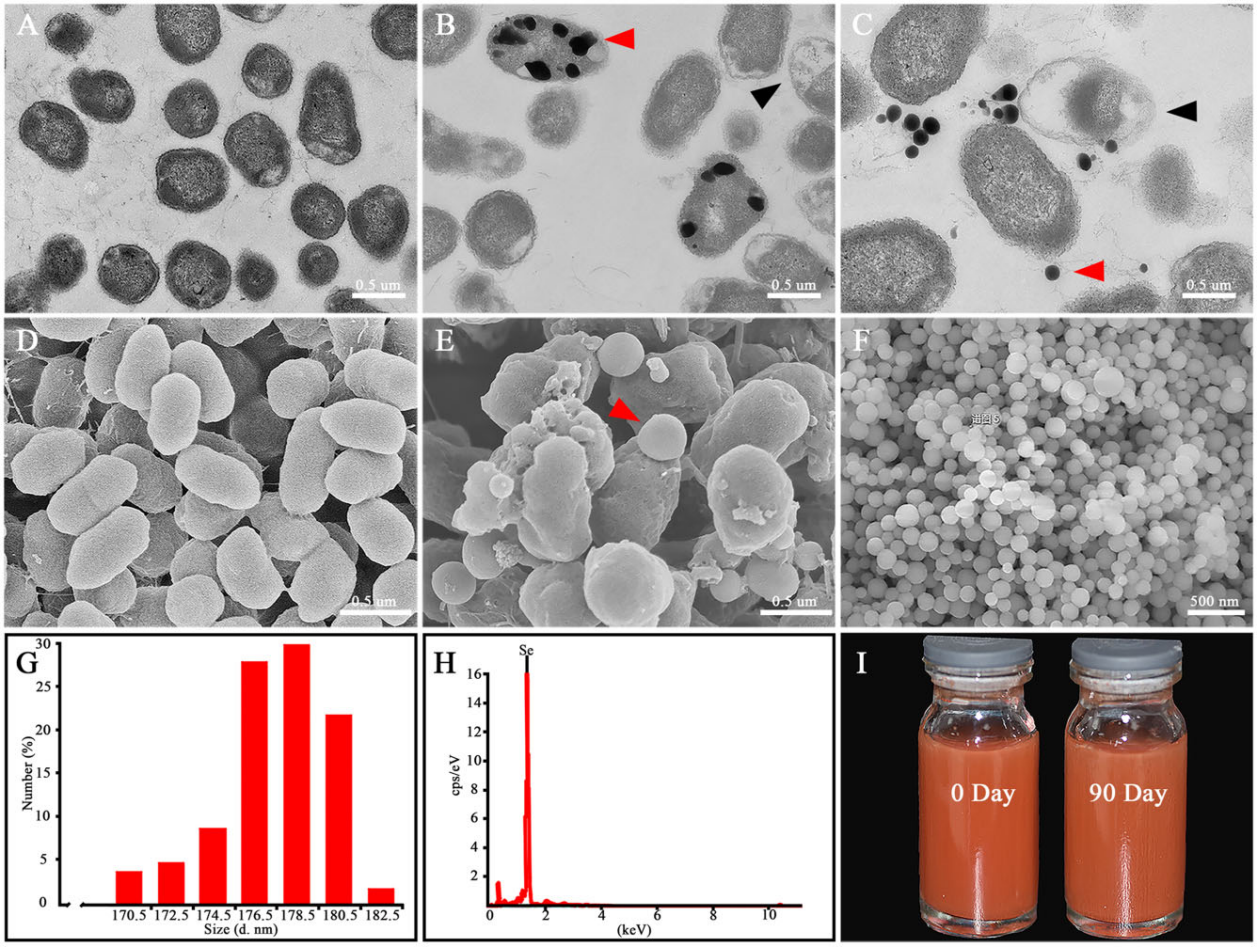
Synthesis and characterization of biogenic SeNPs. (**A**) Transmission electron microscopy images of *P.mirabilis* YC801 grown without and (**B, C**) with Na_2_SeO_3_. (**D**) SEM images of *P.mirabilis* YC801 grown without and (**E**) with Na_2_SeO_3_. (**F**) SEM images of SeNPs. (**G**) DLS spectra of the purified SeNPs. (**H**) Energy dispersive X-ray spectra of the purified SeNPs. (**I**) Direct view of the SeNPs on Days 0 and 90.

The biogenic SeNPs were extracted from *P.mirabilis* YC801 and were then characterized by TEM, SEM, DLS and EDX (Fig. 1). As expected, no nanoparticles were detected without Na_2_SeO_3_ supplementation, as confirmed by TEM and SEM results (Fig. 1A and D). In the presence of Na_2_SeO_3_, the nanoparticles were distributed both intracellularly and extracellularly (Fig. 1B and C). Notably, empty ghost cells, a phenomenon that might result from the SeNPs release from cells, were also detected by TEM (black arrow, Fig. 1C) and confirmed by SEM (red arrow, Fig. 1E). Besides, the purified nanoparticles were found to be spherical (Fig. 1F). DLS results revealed that the nanoparticles had diameters ranging from 170.5 to 182.5 nm, with the average diameter as 178.3 ± 11.5 nm (Fig. 1G). EDX analysis showed that the electron-dense particles formed a peak at 1.37 keV, which is the specific selenium absorption peak [25], demonstrating that the synthesized nanoparticles were indeed SeNPs (Fig. 1H). By comparing the appearance of SeNPs on Days 0 and 90, it was found that biogenic SeNPs exhibited good stability (Fig. 1I).

### Biogenic SeNPs promoted the differentiation and proliferation of NSCs by activating the Wnt/β-Catenin signaling pathway

**Figure 2.**
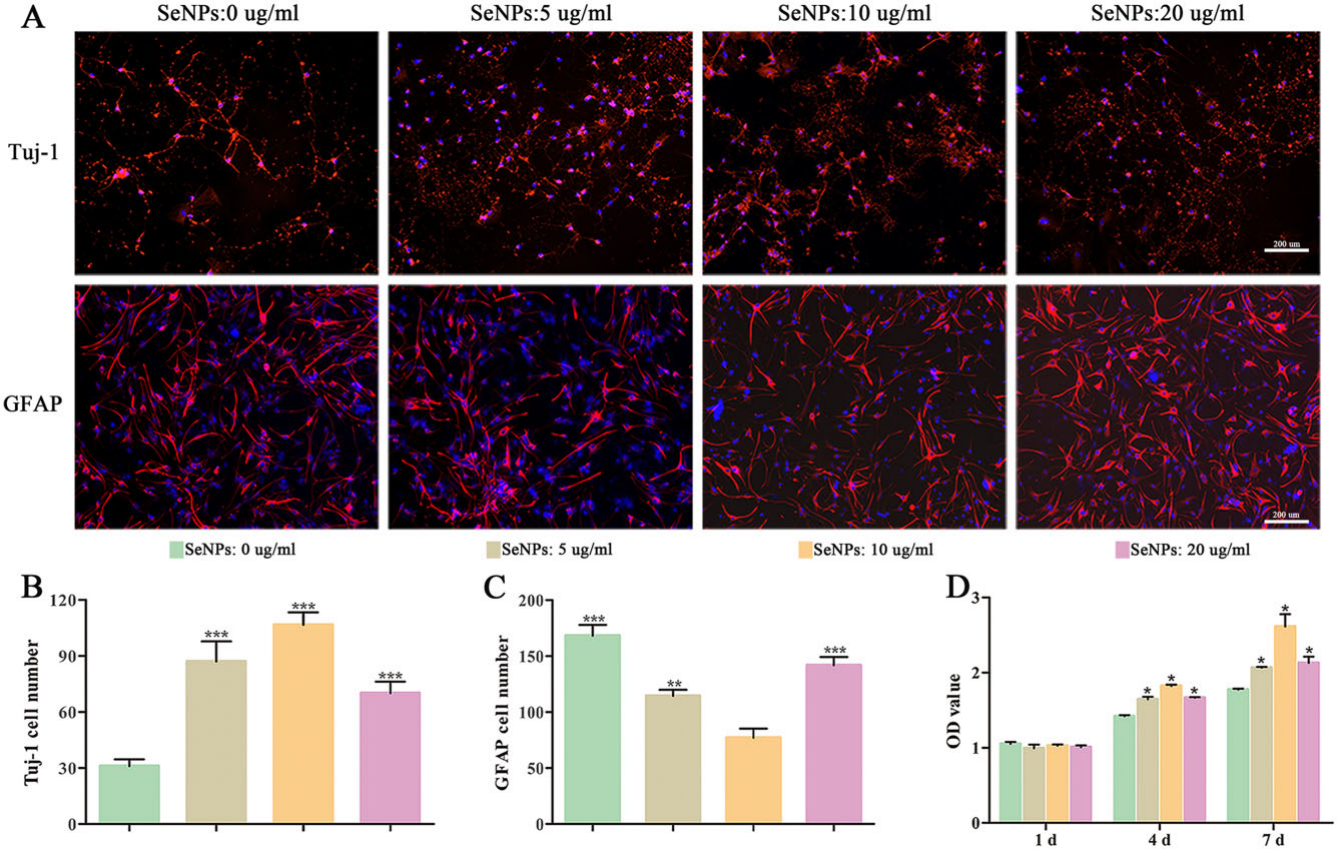
SeNPs promoted the proliferation and differentiation of NSCs. (**A**) NSCs differentiated into neuronal cells and astrocytes at different concentrations of SeNPs. (**B** and **C**) Quantitative analysis in numbers of neuronal cells and astrocytes in each treatment group. (**D**) NSC proliferation via CCK-8 assay at Days 1, 4 and 7 of culture. *P <* *0.05, **P <* *0.01, ***P <* *0.001 by one-way analysis of variance.

SeNPs have been reported to protect nerve cells from neurological disorders [32]. In this study, the influence of SeNPs on the proliferation and differentiation of NSCs was further explored. After treating NSCs with different concentrations of SeNPs at 0, 5, 10 and 20 µg/ml, the immunohistochemistry and CCK-8 assay were implemented to evaluate the impact of SeNPs on NSCs (Fig. 2).

Tuj-1 and GFAP were used as neuronal and astrocyte differentiation markers, respectively. When cultured with 10 µg/ml SeNPs, the number of Tuj-1-positive cells was significantly higher, while the number of GFAP-positive cells was significantly lower than those cultured with 0, 5 and 20 µg/ml SeNPs (Fig. 2A–C), indicating 10 µg/ml as the optimal concentration for SeNPs to promote the differentiation of NSCs into neurons.

On the first day, no significant difference was observed among groups in the absorbance at 450 nm in the CCK-8 assay (Fig. 2D). However, the NSCs proliferation rate in the 10 µg/ml group became significantly higher than the other groups on the third and fifth days, revealing the better biocompatibility of SeNPs at 10 µg/ml concentration.

**Figure 3.**
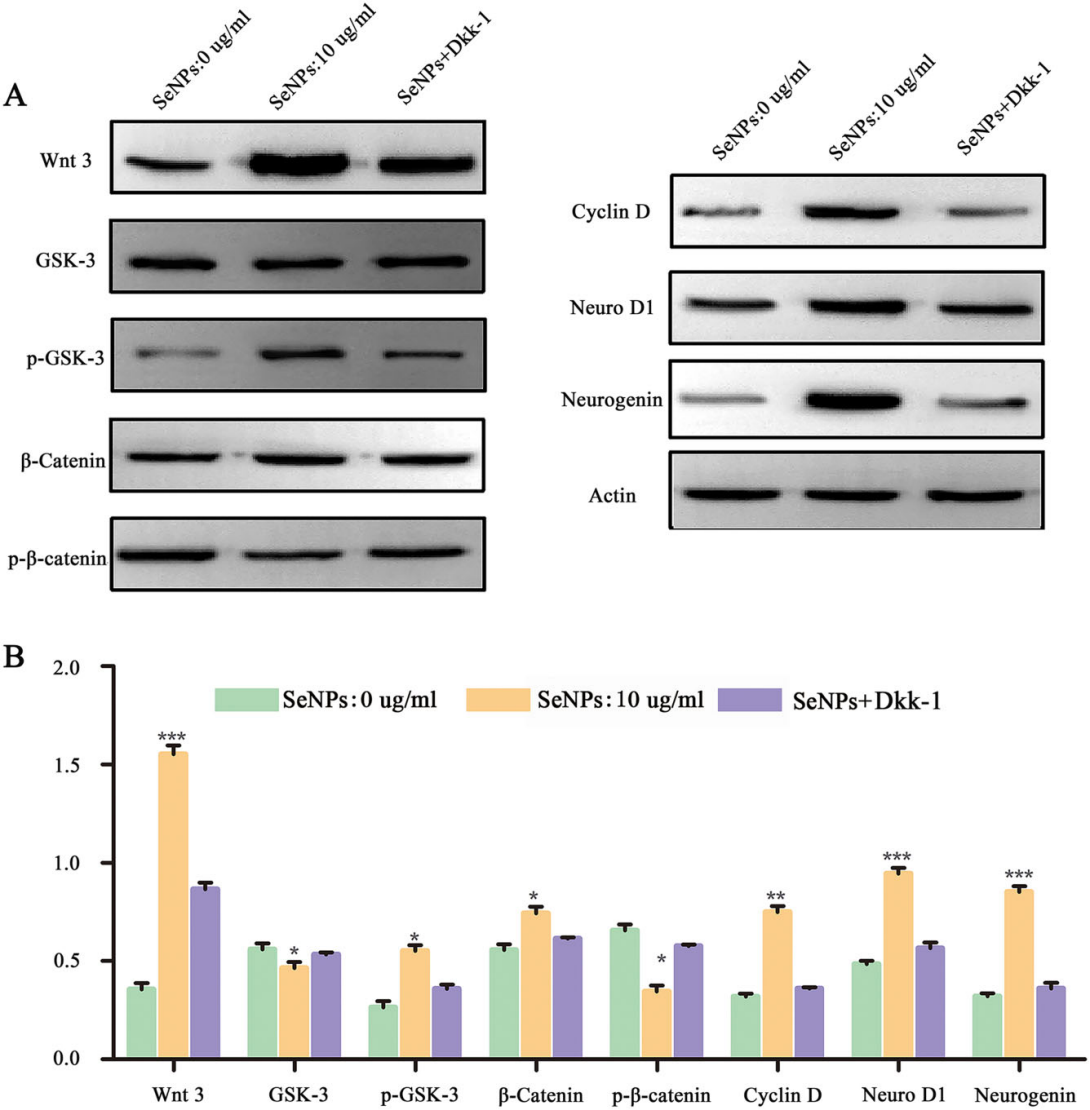
SeNPs regulated the proliferation and differentiation of NSCs via the Wnt/β-Catenin signaling pathway. (**A**) Expression of Wnt3, GSK-3, p-GSK-3, catenin, p-catenin, cyclin D, neuro D1 and neurogenin assessed by western blot. Actin was used as the internal control. (**B**) Quantitative analysis of protein contents among the three groups. *P <* *0.05, **P <* *0.01, ***P <* *0.001 by one-way analysis of variance.

Subsequently, we explored the mechanisms in which SeNPs promoted the proliferation and differentiation of NSCs. Many neuroregulatory pathways are involved in the proliferation and differentiation mechanisms of nerve cells, including the transforming growth factor-β, Notch and Wnt/β-Catenin. The Wnt/β-Catenin signaling pathway is crucial in the nervous centralis neuroepithelium and controls neurogenesis and NSCs renewal [33]. In the Wnt/β-Catenin signaling pathway, Wnt-3 is maintained as a starting protein, and GSK-3 is the main component of the β-caten degradation complex. The wnt/β-catenin signal transduction pathway is involved in the proliferation and differentiation of NSCs by regulating the expression of Cyclin D, Neuro D1 and neurogenin downstream. Dkk-1, the Wnt-3 suppressor, is a potent inhibitor of the Wnt pathway. In the CNS, Dkk-1 increases with age, inhibits neurogenesis and causes cognitive impairment [29, 34]. Increased expressions of Wnt-3, β-catenin and p-GSK-3 in 10 μg/ml SeNPs-treated NSCs were detected compared with the control (0 μg/ml SeNPs) group, and the increased expressions were reversed by the presence of Dkk-1 (SeNPs+Dkk-1 group) (Fig. 3A and B). In contrast, SeNPs significantly reduced the expression of GSK-3. Meanwhile, Cyclin D, neuro D1 and neurogenin expressions significantly increased after SeNPs treatment. The western blot results indicated that the SeNPs mediated the Wnt/β-catenin pathway to promote the proliferation and differentiation of NSCs and enhanced neurogenesis.

### SeNPs reduced tissue damage and improved motor function after SCI

**Figure 4.**
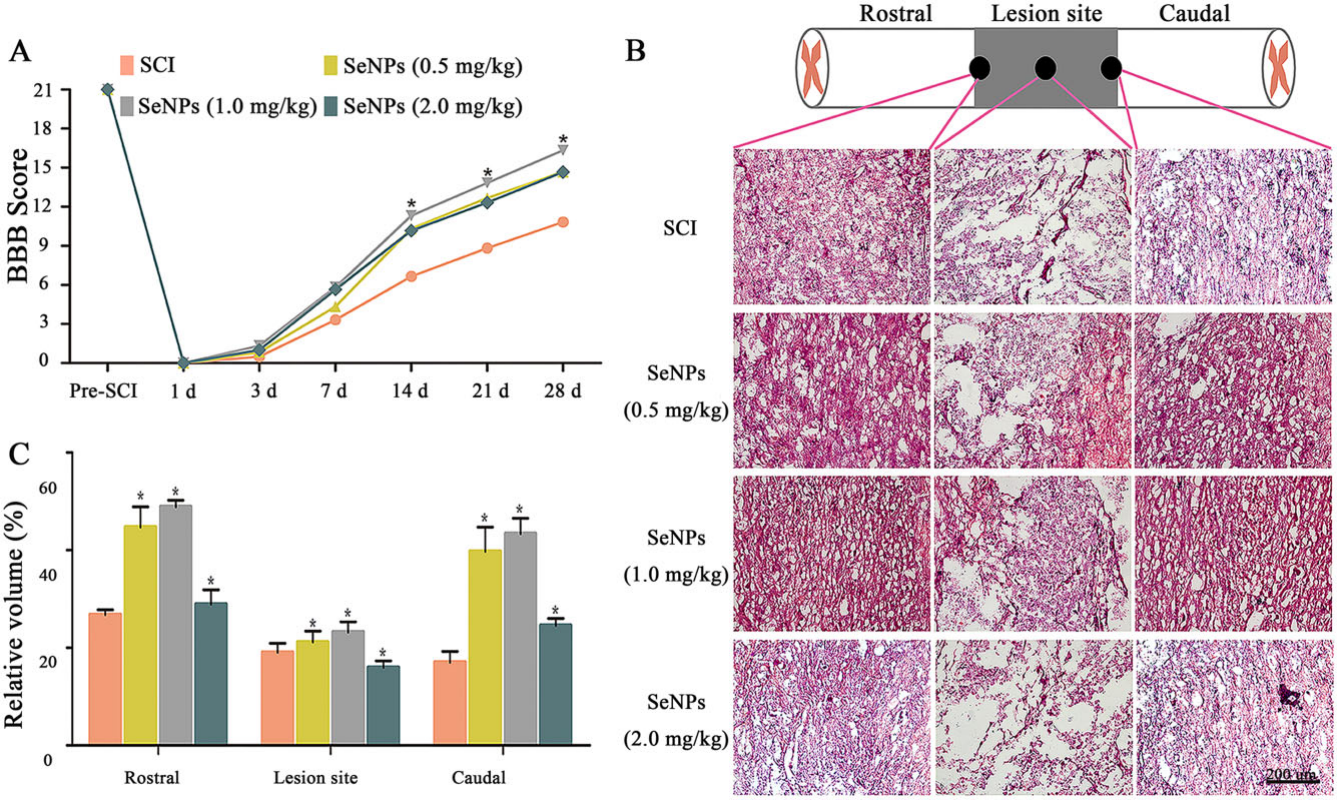
Biogenic SeNPs promoted motor function recovery and ameliorate pathological morphology. (**A**) Motor function of SCI rats was evaluated by the BBB locomotor rating scale on Days 1–28. (**B**) H&E staining of the rostral, lesion site and caudal regions at the injured area. (**C**) Tissue integrity at different sites in each group. *P <* *0.05 by one-way analysis of variance.

BBB locomotor rating scale was performed in SCI rats within 4 weeks postsurgery to assess the motor recovery function of SeNPs (Fig. 4A). All SCI rats showed no observable hind limb movement in the first 3 days. After Day 14, SeNPs-treated animals had higher BBB scores than the SCI group, and this trend became increasingly obvious by Week 4. It is worth noting that the BBB scores in the 1.0 mg/kg SeNPs group were higher than the 0.5 and 2.0 mg/kg groups.

H&E staining was performed 4 weeks post-SCI to explore the histological morphology in the injury site (Fig. 4B and C). H&E staining showed significantly more obvious malformations and cavities in the injured area in the SCI group than in the SeNPs-treated groups, suggesting that SeNPs could reduce cavitation and repair structural disorders, thereby ameliorating the pathological morphology of the injured area. Particularly, improved tissue integrity on the rostral and caudal sides was observed in the SeNPs groups (Fig. 4C). These *in vivo* results demonstrated that SeNPs could enhance functional recovery and reduce tissue damage in rats with SCI.

### Biogenic SeNPs attenuated oxidative stress after SCI

**Figure 5.**
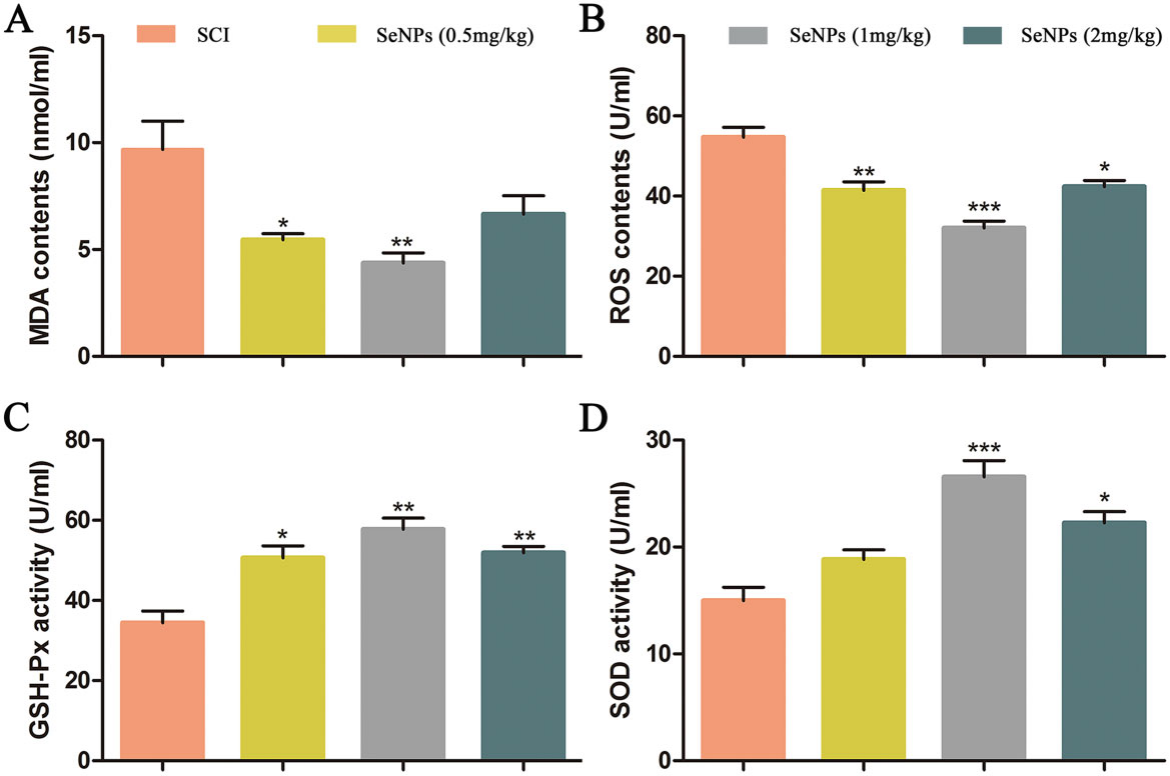
Expression of oxidative stress factors after 14 days of treatment with SeNPs about MDA (**A**), ROS (**B**), GSH-Px (**C**) and SOD (**D**). *P <* *0.05, **P <* *0.01, ***P <* *0.001 by one-way analysis of variance.

After SCI, a high amount of MDA and ROS was produced in the injury site. Over time, GSH-Px and SOD are produced to remove the free radicals and scavenge ROS-induced lipid peroxides to protect the tissue function [35]. To evaluate the inhibitory effects of SeNPs at different concentrations on the oxidative stress of the SCI rats *in vivo*, the expressions of MDA, ROS, SOD, and GSH-Px were detected (Fig. 5). As shown in Fig. 5A and B, MDA and ROS activity increased significantly after SCI, and SeNPs treatment could restore the reduced MDA and ROS activity. The oxidative stress level in the 1.0 mg/kg SeNPs group was significantly lower than that of other SeNPs groups. In contrast, the SOD and GSH-Px contents were higher in the 1.0 mg/kg SeNPs group than in the other groups (Fig. 5C and D), indicating that more antioxidant stress factors were produced. These *in vivo* results demonstrated that SeNPs could improve the oxidative stress microenvironment after SCI and had the potential for SCI treatment.

### Biogenic SeNPs inhibited SCI-induced inflammatory cells and cytokines

**Figure 6.**
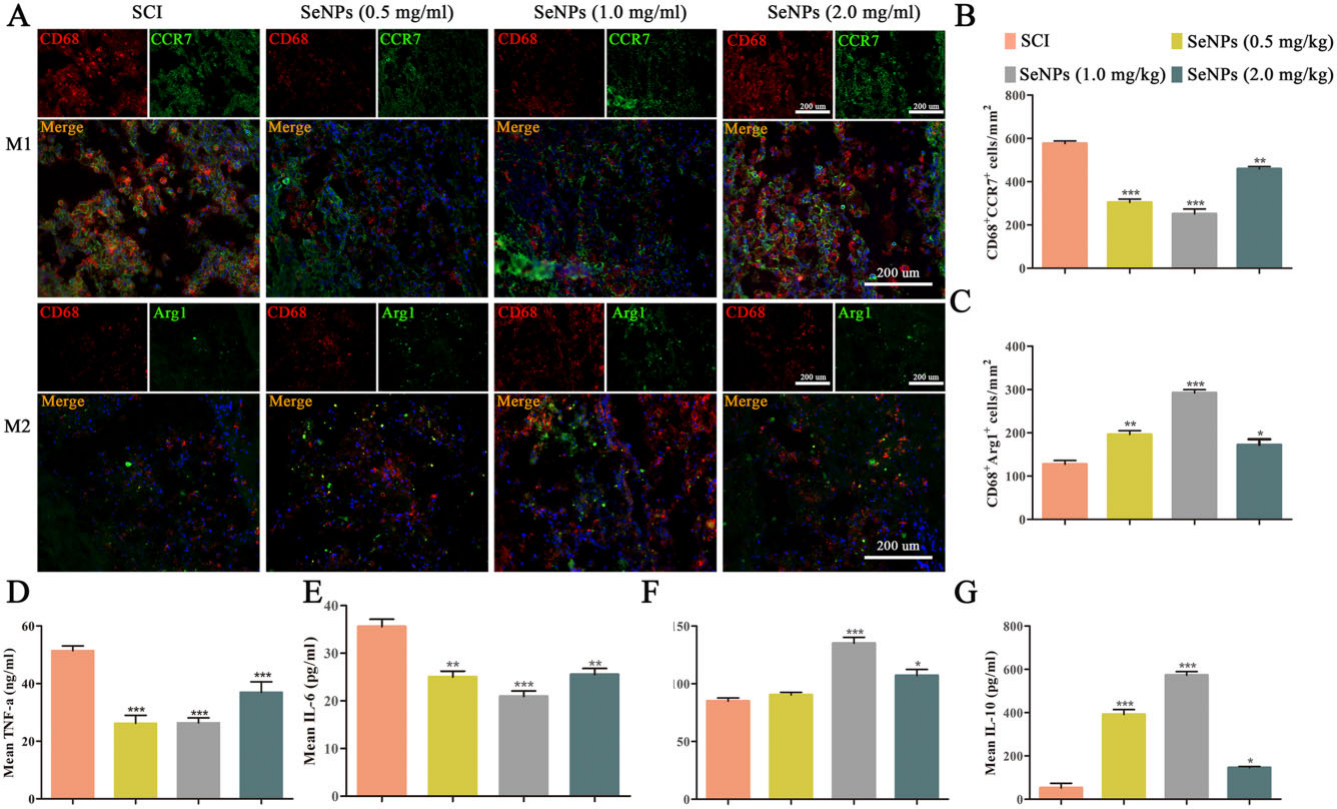
Effects of biogenic SeNPs on M1- and M2-type macrophage polarization in the injured spinal cord and inflammatory cytokine production. (**A**) CD68 and CCR7 for M1 cells, and Arg1 for M2 cells. DAPI staining shows the nuclei. (**B** and **C**) M1 and M2 cell counts in each group. (**D–G**) Expressions of TNF-α, IL-6, IL-4 and IL-6 in each group after 7-day treatment with SeNPs. *P <* *0.05, **P <* *0.01, ***P <* *0.001 by one-way analysis of variance.

The macrophage polarization status was quantified by the immunohistochemical analysis of specific markers for M1-type macrophages (CD68/CCR7) and M2-type macrophages (CD68/Arg1) (Fig. 6A–C). After 7 days, the number of M1-type macrophages in the SCI group was significantly higher, while the number of M2-type macrophage cells was significantly lower than in the SeNPs-treated groups, demonstrating that SCI produced a strong inflammatory response. Among the SeNPs-treated groups, the number of M1-type macrophages in the 1.0 mg/kg SeNPs group was the lowest, whereas the M2-type macrophage number was the highest. It was revealed that SeNPs could regulate the polarization of macrophages and ultimately inhibit the inflammatory response after SCI.

Inflammation levels were measured by inflammatory cytokines (Fig. 6D–G). In the first week, large amounts of proinflammatory factors including TNF-α and IL-6 were detected in the blood of the SCI group (Fig. 6D and E). However, the proinflammatory factor levels in the blood decreased after SeNPs treatment, possibly related to the M2 macrophage polarization. Compared to the 0.5 and 2.0 mg/kg SeNPs groups, a more significant trend of inflammation inhibition could be observed in the 1.0 mg/kg group. Furthermore, an increase in anti-inflammatory factors including IL-4 and IL-10 was observed in the blood (Fig. 6F and G). In addition, the expression of anti-inflammatory factors in the 1.0 mg/kg SeNPs group was higher than those in the 0.5 and 2.0 mg/kg SeNPs group. These results demonstrated that the SeNPs treatment in SCI could effectively ameliorate the damaged SCI microenvironment by reducing the proinflammatory factors and increasing the anti-inflammatory factors.

### Biogenic SeNPs reduced glial scar formation in SCI rats

**Figure 7.**
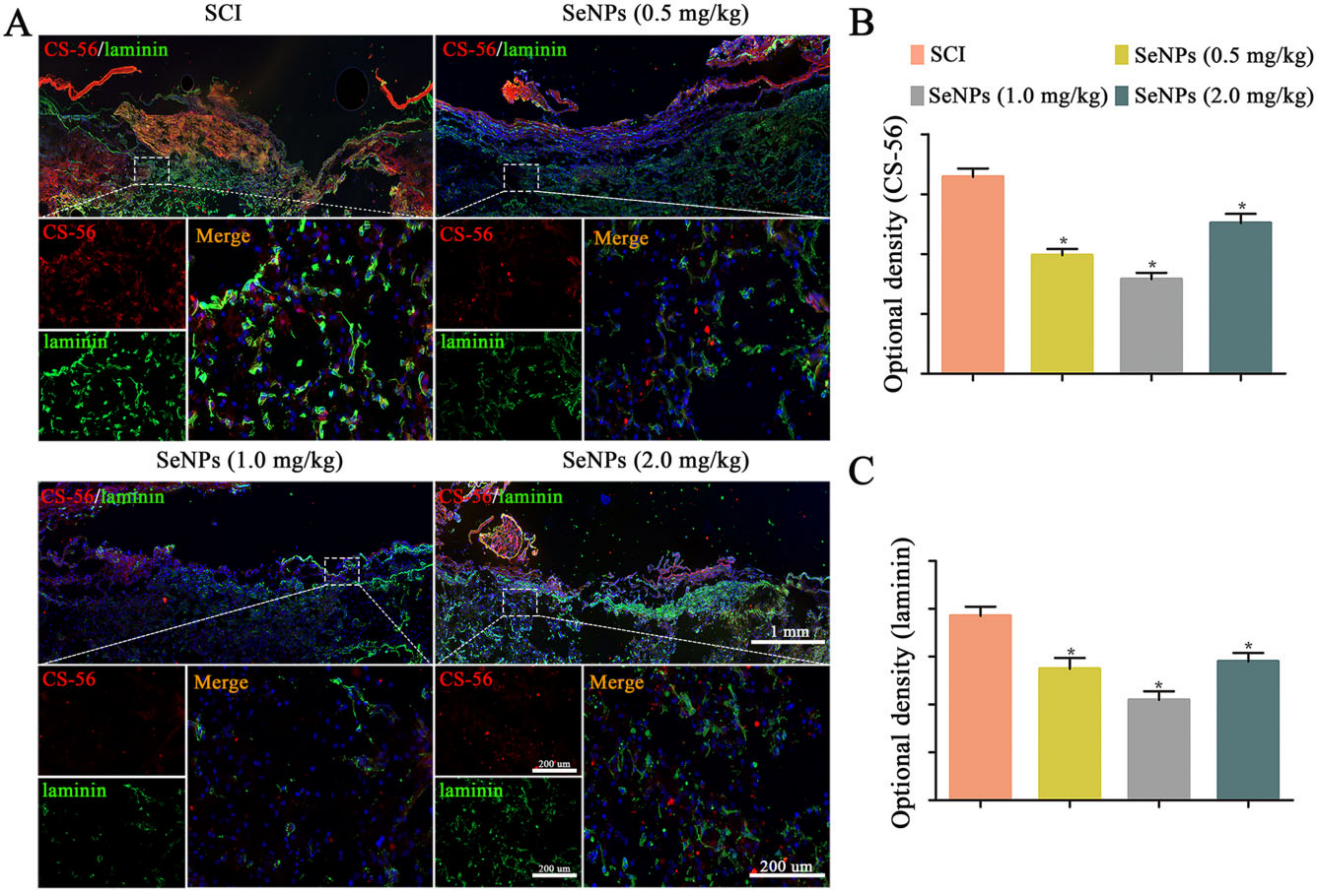
Biogenic SeNPs reduced glial and fibrotic scar formation. (**A**) Chondroitin sulfate (CS-56) and laminin immunofluorescence staining at 4 weeks after injury. (**B** and **C**) Quantitative analysis of the optical density of CS-56 and laminin in each group. *P <* *0.05 by one-way analysis of variance.

In this study, CS-56 and laminin were used as markers of Chondroitin sulfate proteoglycans (CSPGs) and fibrotic glial scars, respectively. Overall, the injury sites of rats in the SCI group were filled with abundant CS-56-positive and laminin-positive staining, whereas those in the SeNPs groups exhibited diminished positive staining (Fig. 7A–C). The fluorescence intensities of CSPGs and fibrotic glial scar-specific proteins at the SCI site were stronger in 0.5 and 2.0 mg/kg SeNPs-treated groups than in the 1.0 mg/kg group, indicating that 1.0 mg/kg SeNPs treatment could lead to the strongest inhibition of glial scar formation relevant for neuronal function. These results indicated that SeNPs treatment could provide an appropriate microenvironment for axon regeneration.

### Biogenic SeNPs promoted the neurocyte regeneration and functional axonal regeneration

**Figure 8.**
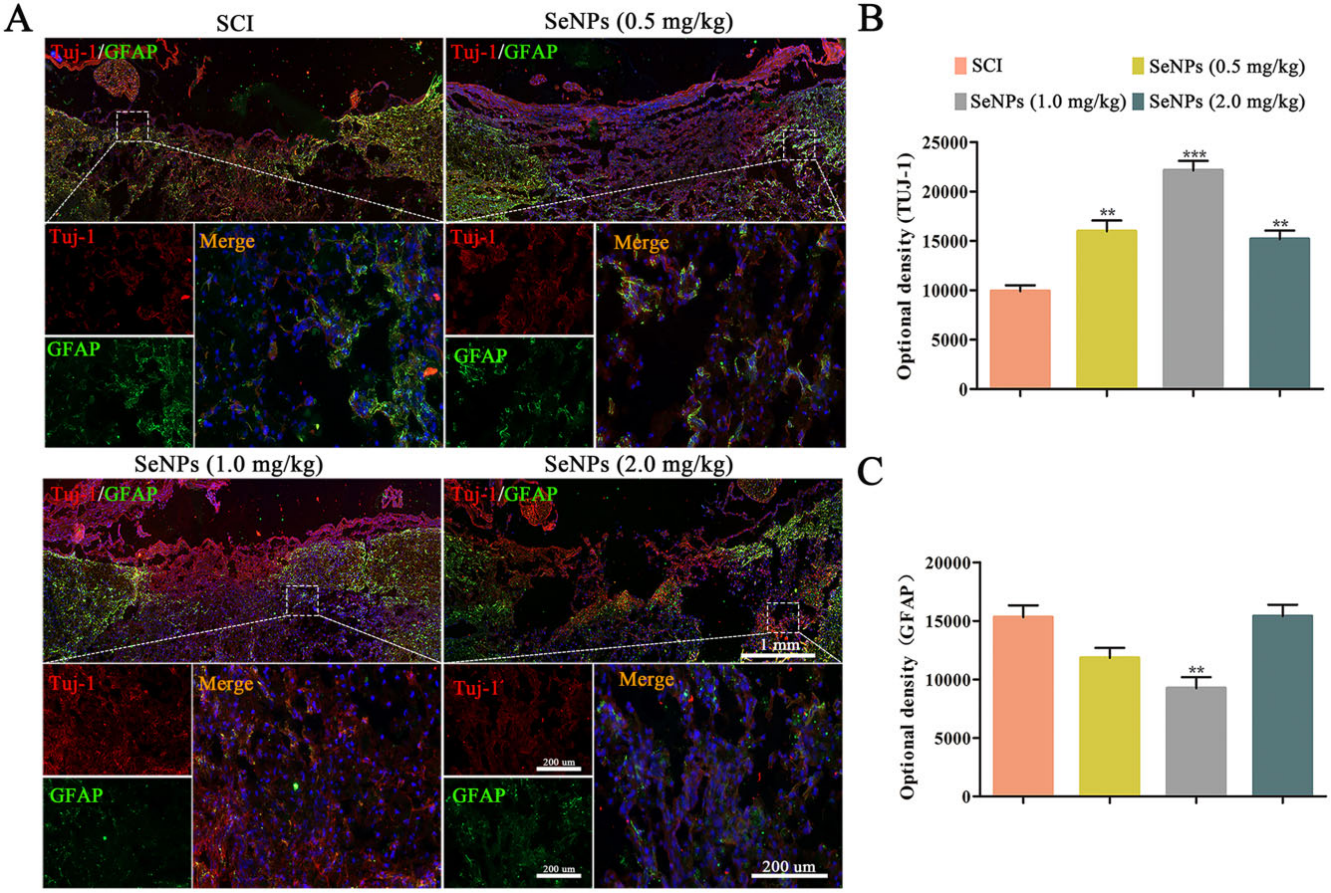
Biogenic SeNPs promoted neuronal differentiation. (**A**) Beta-tubulin III (Tuj-1) and GFAP expression at 4 weeks after injury. (**B** and **C**) Quantitative analysis of the optical densities of Tuj-1 and GFAP. **P <* *0.01, ***P <* *0.001 by one-way analysis of variance.

Double-immunofluorescence stainings for Tuj-1 and GFAP were performed to identify newly formed neurons and astrocytes at the SCI site, respectively. A lower Tuj-1 expression was observed in the lesion site of the spinal cord in the SCI group (Fig. 8A and B), whereas Tuj-1 expression was significantly increased in the SeNPs-treated groups. The Tuj-1 expression was also significantly higher in the 1.0 mg/kg SeNPs than in other groups. The formation of astrocytes indicated by the GFAP marker was inhibited by the SeNPs, and its amount was the lowest in rats treated with 1.0 mg/kg SeNPs (Fig. 8A and C).

**Figure 9.**
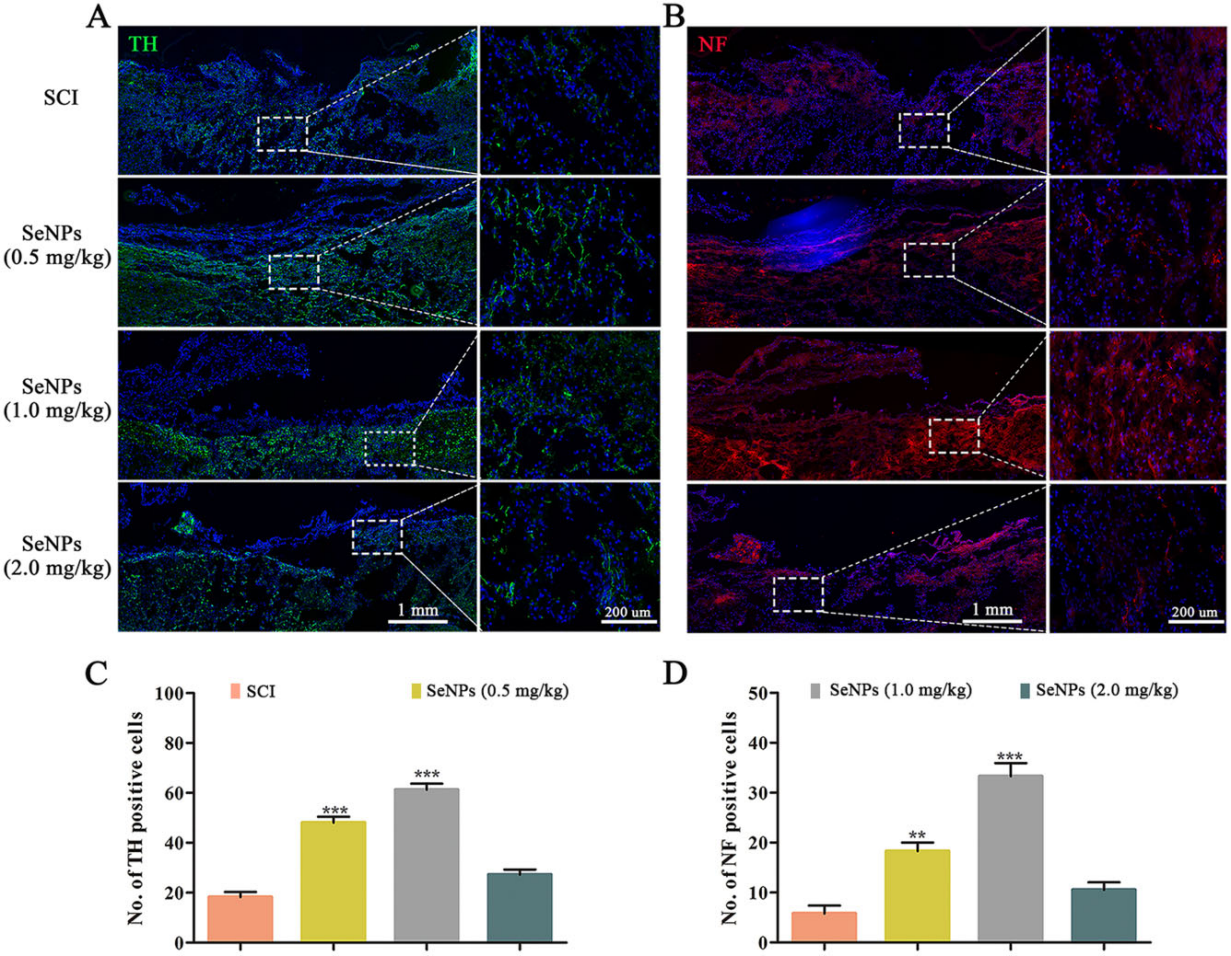
Biogenic SeNPs promoted motor function-related axon regeneration. (**A**) TH-positive neurons in the different groups at the injury site. (**B**) Regenerated NFs at injury sites in each group at 4 weeks postinjury. (**C** and **D**) The number of TH-positive and NF-positive neurons at the injury site in each group. **P <* *0.01, ***P <* *0.001 by one-way analysis of variance.

Serotonin (5-HT) is a crucial neurotransmitter that affects motor function, and its synthesis is catalyzed by TH [36]. NF is highly specific for nerve cell injury and death and is used as a biomarker for axonal nerve repair [37]. Hence, TH and NF staining were used to detect motor function and nerve recovery in the injured site (Fig. 9A and B). The TH-positive and NF-positive cells are significantly higher in the SeNPs groups, while a few immunofluorescences could be observed in the injury sites in the SCI group. Consistent with the previous results, the regeneration of serotonergic axons and axon myelination showed the best curative effect at the SeNPs concentration of 1.0 mg/kg (Fig. 9C and D).

## Discussion

SCI-induced inflammation and oxidative stress are highly disordered metabolic processes, mainly manifested as an imbalance of the microenvironment, ultimately leading to injury recovery and progressive tissue degradation [15, 38, 39]. Macrophages play a vital role in tissue repair in the inflammatory microenvironment of SCI, but this process often results in a state where the damaged tissue cannot be repaired due to the change of macrophage polarization direction. In addition, the inflammation process is often accompanied by the participation of oxidative stress in neurological diseases [16, 19]. As one of the main harmful effects of secondary injury, the ROS level will continue to increase [31, 40–42].

SeNPs, a new form of selenium with low toxicity and high biocompatibility, have been reported to possess neuroprotective properties, including anti-inflammation and antioxidative stress in different animal models of neurological diseases [9]. The therapeutic effect of SeNPs is related to the particle diameter and dosage [43, 44]. In this study, the SeNPs produced by *P.mirabilis* YC801 had a diameter of 178.3 ± 11.5 nm (Fig. 1). Studies have found that SeNPs showed comparable safety at the diameter of 25–80 and 50–250 nm, especially at the diameter of <200 nm, and they could directly scavenge free radicals *in vitro* since the specific surface area could provide sufficient active sites [45, 46]. In addition, the levels of liver ROS and MDA in mice showed varying degrees of decline with different doses of SeNPs (1 and 4 mg/kg) [43]. The SeNPs possess more antioxidative and retentive potential at a 0.5 mg/kg concentration *in vivo* [47]. Moreover, SeNPs had no cytotoxicity on the growth and proliferation of human normal epithelial cells (NCM460) at concentrations below 25 µg/ml *in vitro* [48]. But as a neuroprotective agent with multiple functions, the specific roles of SeNPs in SCI remain unclear. Therefore, this study aimed to explore the protective effect of SeNPs on functional recovery after SCI and its underlying mechanism. Hence, different SeNPs concentrations were used for experiments, and the SeNPs exhibited optimal anti-inflammatory and antioxidant stress effects at concentrations of 10 µg/ml and 1.0 mg/kg *in vitro* and *in vivo*, respectively.

NSCs have great potential in treating CNS diseases [28]. There is increasing evidence showing that NSCs mainly differentiate into astrocytes rather than neurons due to the deterioration of the microenvironment after SCI, and astrocytes then form glial scars in the late stages, which limits nerve repair [49]. Therefore, it is essential to create a microenvironment that induces NSCs to differentiate into neurons for SCI treatment. SeNPs are associated with CNS diseases. For example, treating HD with SeNPs could significantly reduce neuronal death and alleviate behavioral disorders [11]. In this study, CCK-8 and cell immunofluorescence results indicated that SeNPs increased the neuronal proliferation and differentiation of NSCs markedly (Fig. 2). Wnt/β-catenin signaling pathway, which plays a critical effect in the survival, proliferation and self-renewal of stem cells, is related to SeNPs-triggered proliferation and differentiation of NSCs (Fig. 3). It is worth mentioning that the differentiation of astrocytes increases at a SeNPs concentration of over 10 µg/ml, suggesting that more glial scar formation inhibits nerve repair in the SCI treatment.

*In vivo*, the behavioral test revealed that SeNPs promoted functional motor recovery after SCI (Fig. 4A), indicating the chaotic microenvironment was improved after SeNPs treatment.

Oxidative stress caused by excessive ROS production has become an important mechanism in the progression of SCI [39]. SeNPs could reduce the tissue damage caused by oxidative stress in mice by inhibiting the process of ROS generation [50]. Moreover, GSH-Px from SeNPs can be an antioxidant and effectively protect cells from oxidative stress [51]. Therefore, suppressing oxidative stress has been considered an effective therapeutic strategy to improve SCI recovery. The SeNPs can not only inhibit the production of ROS and MDA but also restore the activity of SOD and GSH-Px in SCI (Fig. 5).

Excessive ROS can also activate macrophages, causing inflammation and aggravating the progression of SCI [17]. The effects of SeNPs on inflammation in SCI rats were further investigated. After SCI, activated macrophages can not only release proinflammatory cytokines that cause cytotoxicity and demyelination but also produce neuroprotective molecules that release anti-inflammatory factors and stimulate axon regeneration and sprouting [52]. According to the role of macrophages in SCI, they are divided into two subpopulations: M1 cells that release proinflammatory cytokines and M2 cells that release anti-inflammatory cytokines [53]. Therefore, phase change between M1 and M2 polarization will lead to a shift in inflammation balance [54]. In this study, M1-types macrophages were the main infiltrating macrophages in the SCI group (Fig. 6). In terms of macrophage polarization, SeNPs can not only significantly lower M1-type macrophages but also notably enhance M2-type macrophages after SCI. Additionally, SeNPs can inhibit the proinflammatory factors including TNF-α and IL-1β, and promote the levels of anti-inflammatory factors including IL-4 and IL-10. Therefore, SeNPs can diminish M1-type microglia and ameliorate M2-type microglia by targeting the macrophages after SCI.

CSPGs secreted by the astrocytes in the injury site have been regarded as a chemical barrier to block the growth of axonal regeneration and functional recovery. Other fibrotic scars are produced by fibroblasts as the excessive deposition of extracellular matrix [41]. Glial scars formed by astrocytes after SCI inhibit the expansion of the inflammatory microenvironment [49]. However, a prominent and permanent glial scar generates a dense barrier that inhibits axon regeneration at the lesion site [55]. Thus, finding an intervention that reduces the glial scar area to benefit axon regeneration and remyelination is critical for functional recovery in treating SCI. After SeNPs treatment, the formation of glial scars is inhibited. In addition, the fibrotic component areas of the glial scar were also reduced (Fig. 7). These results revealed that SeNPs treatment could subsequently provide an appropriate microenvironment for axon regeneration.

The impacts of SeNPs on the differentiation of NSCs were demonstrated in our previous studies. Hence, GFAP and Tuj-1 double labeling was performed to detect the fibrotic component areas of the axon regeneration at the lesion site of SCI. Sufficient regenerated neurons and a small number of astrocytes were detected in the injured sites with the SeNPs treatment, which was related to the dose of SeNPs (Fig. 8). Furthermore, neural degeneration is the major cause of dysfunction after SCI [56]. The regeneration of TH and NF is related to the recovery of motor function [36, 37, 57]. In this study, TH and NF-positive nerve fibers were discovered in the SeNPs group (Fig. 9). These results indicated that SeNPs facilitated axonal regrowth and promoted the regeneration of functional neurons and axons in the SCI site.

## Conclusion

In this study, a novel biogenic SeNPs derived from *P.mirabilis* YC801 was successfully constructed. *In vitro* studies showed that the biogenic SeNPs regulated the proliferation and differentiation of NSCs by activating the Wnt/β-Catenin signaling pathway. Interestingly, SeNPs could promote the directed differentiation of NSCs into neural cells and inhibit the generation of astrocytes. *In vivo* studies demonstrated that SeNPs could help SCI rats recover motor function, inhibit the damage at injury sites, decrease oxidative stress and inflammatory response, reduce glial scar formation and promote the regeneration of serotonergic and dopaminergic axons. To conclude, bioderived SeNPs may be a promising therapeutic approach for SCI by oral administration. Further research is needed to better understand the underlying mechanism by which SeNPs mediate the proliferation and differentiation of NSCs, which will lay a foundation for future research on nerve repair in SCI.

## Funding

This study was supported by the Key Program of the Anhui Educational Committee (Grant No. KJ2020ZD51 and KJ2021ZD0089), Innovation Fund of Spinal Deformity Clinical Research Center of Anhui Province (Grant No. AHJZJX-GG2022-001), 512 Talents Development Project of Bengbu Medical College (Grant No. by51202302 and by51202309), Distinguished Young Scholars of First Affiliated Hospital of Bengbu Medical College (Grant No. 2021byyfyjq01) and Science Research Project of Bengbu Medical College (Grant No. 2021bypd006).

*Conflicts* *of interest statement*. None declared.

## References

[1] El-Ghazaly MA , Fadel N, Rashed E, El-Batal A, Kenawy SA. Anti-inflammatory effect of selenium nanoparticles on the inflammation induced in irradiated rats. Can J Physiol Pharmacol 2017;95:101–10. 27936913 10.1139/cjpp-2016-0183

[2] Vinceti M , Mandrioli J, Borella P, Michalke B, Tsatsakis A, Finkelstein Y. Selenium neurotoxicity in humans: bridging laboratory and epidemiologic studies. Toxicol Lett 2014;230:295–303. 24269718 10.1016/j.toxlet.2013.11.016

[3] Rayman MP. Selenium and human health. Lancet 2012;379:1256–68. 22381456 10.1016/S0140-6736(11)61452-9

[4] Zhang J , Wang X, Xu T. Elemental selenium at nano size (Nano-Se) as a potential chemopreventive agent with reduced risk of selenium toxicity: comparison with se-methylselenocysteine in mice. Toxicol Sci 2008;101:22–31. 17728283 10.1093/toxsci/kfm221

[5] Hosnedlova B , Kepinska M, Skalickova S, Fernandez C, Ruttkay-Nedecky B, Peng Q, Baron M, Melcova M, Opatrilova R, Zidkova J, Bjorklund G, Sochor J, Kizek R. Nano-selenium and its nanomedicine applications: a critical review. Int J Nanomedicine 2018;13:2107–28. 29692609 10.2147/IJN.S157541 PMC5901133

[6] Huang B , Zhang J, Hou J, Chen C. Free radical scavenging efficiency of Nano-Se in vitro. Free Radic Biol Med 2003;35:805–13. 14583345 10.1016/s0891-5849(03)00428-3

[7] Khurana A , Tekula S, Saifi MA, Venkatesh P, Godugu C. Therapeutic applications of selenium nanoparticles. Biomed Pharmacother 2019;111:802–12. 30616079 10.1016/j.biopha.2018.12.146

[8] Liu H , Zhang W, Fang Y, Yang H, Tian L, Li K, Lai W, Bian L, Lin B, Liu X, Xi Z. Neurotoxicity of aluminum oxide nanoparticles and their mechanistic role in dopaminergic neuron injury involving p53-related pathways. J Hazard Mater 2020;392:122312. 32105957 10.1016/j.jhazmat.2020.122312

[9] Sun J , Wei C, Liu Y, Xie W, Xu M, Zhou H, Liu J. Progressive release of mesoporous nano-selenium delivery system for the multi-channel synergistic treatment of Alzheimer’s disease. Biomaterials 2019;197:417–31. 30638753 10.1016/j.biomaterials.2018.12.027

[10] Ali H , El-Sayed NM, Khodeer DM, Ahmed A, Hanna PA, Moustafa Y. Nano selenium ameliorates oxidative stress and inflammatory response associated with cypermethrin-induced neurotoxicity in rats. Ecotoxicol Environ Saf 2020;195:110479. 32199213 10.1016/j.ecoenv.2020.110479

[11] Cong W , Bai R, Li YF, Wang L, Chen C. Selenium nanoparticles as an efficient nanomedicine for the therapy of Huntington’s disease. ACS Appl Mater Interfaces 2019;11:34725–35. 31479233 10.1021/acsami.9b12319

[12] Liu X , Zhang Y, Wang Y, Qian T. Inflammatory response to spinal cord injury and its treatment. World Neurosurg 2021;155:19–31. 34375779 10.1016/j.wneu.2021.07.148

[13] Silvestro S , Bramanti P, Trubiani O, Mazzon E. Stem cells therapy for spinal cord injury: an overview of clinical trials. Int J Mol Sci 2020;21:659. 10.3390/ijms21020659 PMC7013533 31963888

[14] Orr MB , Gensel JC. Spinal cord injury scarring and inflammation: therapies targeting glial and inflammatory responses. Neurotherapeutics 2018;15:541–53. 29717413 10.1007/s13311-018-0631-6 PMC6095779

[15] Chen YQ , Wang SN, Shi YJ, Chen J, Ding SQ, Tang J, Shen L, Wang R, Ding H, Hu JG, Lu HZ. CRID3, a blocker of apoptosis associated speck like protein containing a card, ameliorates murine spinal cord injury by improving local immune microenvironment. J Neuroinflammation 2020;17:255. 32861243 10.1186/s12974-020-01937-8 PMC7456508

[16] Milich LM , Ryan CB, Lee JK. The origin, fate, and contribution of macrophages to spinal cord injury pathology. Acta Neuropathol 2019;137:799–800. 30929040 10.1007/s00401-019-01992-3 PMC6510275

[17] He L , Huang G, Liu H, Sang C, Liu X, Chen T. Highly bioactive zeolitic imidazolate framework-8-capped nanotherapeutics for efficient reversal of reperfusion-induced injury in ischemic stroke. Sci Adv 2020;6:eaay9751. 32206718 10.1126/sciadv.aay9751 PMC7080448

[18] Bloom O , Herman PE, Spungen AM. Systemic inflammation in traumatic spinal cord injury. Exp Neurol 2020;325:113143. 31843491 10.1016/j.expneurol.2019.113143

[19] Gensel JC , Zhang B. Macrophage activation and its role in repair and pathology after spinal cord injury. Brain Res 2015;1619:1–11. 25578260 10.1016/j.brainres.2014.12.045

[20] Fu S , Lv R, Wang L, Hou H, Liu H, Shao S. Resveratrol, an antioxidant, protects spinal cord injury in rats by suppressing MAPK pathway. Saudi J Biol Sci 2018;25:259–66. 29472775 10.1016/j.sjbs.2016.10.019 PMC5815991

[21] Yao X , Sun C, Fan B, Zhao C, Zhang Y, Duan H, Pang Y, Shen W, Li B, Wang X, Liu C, Zhou H, Kong X, Feng S. Neurotropin exerts neuroprotective effects after spinal cord injury by inhibiting apoptosis and modulating cytokines. J Orthop Translat 2021;26:74–83. 33437626 10.1016/j.jot.2020.02.011 PMC7773959

[22] Rossignol S , Schwab M, Schwartz M, Fehlings MG. Spinal cord injury: time to move? J Neurosci 2007;27:11782–92. 17978014 10.1523/JNEUROSCI.3444-07.2007 PMC6673354

[23] Jeong HJ , Yun Y, Lee SJ, Ha Y, Gwak SJ. Biomaterials and strategies for repairing spinal cord lesions. Neurochem Int 2021;144:104973. 33497713 10.1016/j.neuint.2021.104973

[24] Bai K , Hong B, He J, Huang W. Antioxidant capacity and hepatoprotective role of chitosan-stabilized selenium nanoparticles in concanavalin A-induced liver injury in mice. Nutrients 2020;12:857. 10.3390/nu12030857 PMC7146609 32210138

[25] Ali F , Saeed K, Fatemeh H. Nano-bio selenium synthesized by *Bacillus subtilis* modulates broiler performance, intestinal morphology and microbiota, and expression of tight junction's proteins. Biol Trace Elem Res 2022;200:1811–25. 34075493 10.1007/s12011-021-02767-2

[26] Wang Y , Shu X, Hou J, Lu W, Zhao W, Huang S, Wu L. Selenium nanoparticle synthesized by *Proteus mirabilis* YC801: an efficacious pathway for selenite biotransformation and detoxification. Int J Mol Sci 2018;19:3809. 10.3390/ijms19123809 PMC6321198 30501097

[27] Guan B , Yan R, Li R, Zhang X. Selenium as a pleiotropic agent for medical discovery and drug delivery. Int J Nanomedicine 2018;13:7473–90. 30532534 10.2147/IJN.S181343 PMC6241719

[28] Zhou P , Xu P, Guan J, Zhang C, Chang J, Yang F, Xiao H, Sun H, Zhang Z, Wang M, Hu J, Mao Y. Promoting 3D neuronal differentiation in hydrogel for spinal cord regeneration. Colloids Surf B Biointerfaces 2020;194:111214. 32599502 10.1016/j.colsurfb.2020.111214

[29] Tiwari SK , Agarwal S, Seth B, Yadav A, Nair S, Bhatnagar P, Karmakar M, Kumari M, Chauhan LK, Patel DK, Srivastava V, Singh D, Gupta SK, Tripathi A, Chaturvedi RK, Gupta KC. Curcumin-loaded nanoparticles potently induce adult neurogenesis and reverse cognitive deficits in Alzheimer's disease model via canonical Wnt/beta-catenin pathway. ACS Nano 2014;8:76–103. 24467380 10.1021/nn405077y

[30] Duan FX , Shi YJ, Chen J, Ding SQ, Wang FC, Tang J, Wang R, Shen L, Xi J, Qi Q, Lu HZ, Hu JG. Neuroprotective effects of P7C3 against spinal cord injury in rats. Exp Biol Med (Maywood) 2019;244:1680–7. 31718264 10.1177/1535370219888620 PMC6963372

[31] Jiang L , Wei ZC, Xu LL, Yu SY, Li C. Inhibition of miR-145-5p reduces spinal cord injury-induced inflammatory and oxidative stress responses via affecting Nurr1-TNF-alpha signaling axis. Cell Biochem Biophys 2021;79:791–9. 34133012 10.1007/s12013-021-00992-z

[32] Ibrahim HM , Zommara MA, Elnaggar ME. Ameliorating effect of selenium nanoparticles on cyclophosphamide-induced hippocampal neurotoxicity in male rats: light, electron microscopic and immunohistochemical study. Folia Morphol (Warsz) 2021;80:806–19. 33084015 10.5603/FM.a2020.0117

[33] Hadrup N , Loeschner K, Mandrup K, Ravn-Haren G, Frandsen HL, Larsen EH, Lam HR, Mortensen A. Subacute oral toxicity investigation of selenium nanoparticles and selenite in rats. Drug Chem Toxicol 2019;42:76–83. 30032689 10.1080/01480545.2018.1491589

[34] Seib DR , Corsini NS, Ellwanger K, Plaas C, Mateos A, Pitzer C, Niehrs C, Celikel T, Martin-Villalba A. Loss of Dickkopf-1 restores neurogenesis in old age and counteracts cognitive decline. Cell Stem Cell 2013;12:204–14. 23395445 10.1016/j.stem.2012.11.010

[35] Hou Y , Luan J, Huang T, Deng T, Li X, Xiao Z, Zhan J, Luo D, Hou Y, Xu L, Lin D. Tauroursodeoxycholic acid alleviates secondary injury in spinal cord injury mice by reducing oxidative stress, apoptosis, and inflammatory response. J Neuroinflammation 2021;18:216. 34544428 10.1186/s12974-021-02248-2 PMC8454169

[36] Han Q , Feng J, Qu Y, Ding Y, Wang M, So KF, Wu W, Zhou L. Spinal cord maturation and locomotion in mice with an isolated cortex. Neuroscience 2013;253:235–44. 24012835 10.1016/j.neuroscience.2013.08.057

[37] Gafson AR , Barthelemy NR, Bomont P, Carare RO, Durham HD, Julien JP, Kuhle J, Leppert D, Nixon RA, Weller RO, Zetterberg H, Matthews PM. Neurofilaments: neurobiological foundations for biomarker applications. Brain 2020;143:1975–98. 32408345 10.1093/brain/awaa098 PMC7363489

[38] Chen J , Chen YQ, Shi YJ, Ding SQ, Shen L, Wang R, Wang QY, Zha C, Ding H, Hu JG, Lu HZ. VX-765 reduces neuroinflammation after spinal cord injury in mice. Neural Regen Res 2021;16:1836–47. 33510091 10.4103/1673-5374.306096 PMC8328782

[39] Ding LZ , Xu J, Yuan C, Teng X, Wu QM. MiR-7a ameliorates spinal cord injury by inhibiting neuronal apoptosis and oxidative stress. Eur Rev Med Pharmacol Sci 2020;24:11–7. 31957813 10.26355/eurrev_202001_19890

[40] Liu G , Fan G, Guo G, Kang W, Wang D, Xu B, Zhao J. FK506 attenuates the inflammation in rat spinal cord injury by inhibiting the activation of NF-κB in microglia cells. Cell Mol Neurobiol 2017;37:843–55. 27572744 10.1007/s10571-016-0422-8 PMC11482064

[41] Anjum A , Yazid MD, Fauzi DM, Idris J, Ng A, Selvi NA, Ismail O, Athi KR, Lokanathan Y. Spinal cord injury: pathophysiology, multimolecular interactions, and underlying recovery mechanisms. Int J Mol Sci 2020;21:7533. 10.3390/ijms21207533 PMC7589539 33066029

[42] Patel NP , Huang JH. Hyperbaric oxygen therapy of spinal cord injury. Med Gas Res 2017;7:133–43. 28744367 10.4103/2045-9912.208520 PMC5510295

[43] Kondaparthi P , Deore M, Naqvi S, Flora S. Dose-dependent hepatic toxicity and oxidative stress on exposure to nano and bulk selenium in mice. Environ Sci Pollut Res Int 2021;28:53034–44. 34023997 10.1007/s11356-021-14400-9

[44] Peng D , Zhang J, Liu Q, Taylor EW. Size effect of elemental selenium nanoparticles (Nano-Se) at supranutritional levels on selenium accumulation and glutathione S-transferase activity. J Inorg Biochem 2007;101:1457–63. 17664013 10.1016/j.jinorgbio.2007.06.021

[45] Cai W , Hu T, Bakry AM, Zheng Z, Xiao Y, Huang Q. Effect of ultrasound on size, morphology, stability and antioxidant activity of selenium nanoparticles dispersed by a hyperbranched polysaccharide from *Lignosus rhinocerotis*. Ultrason Sonochem 2018;42:823–31. 29429736 10.1016/j.ultsonch.2017.12.022

[46] Mal J , Veneman WJ, Nancharaiah YV, van Hullebusch ED, Peijnenburg WJ, Vijver MG, Lens PN. A comparison of fate and toxicity of selenite, biogenically, and chemically synthesized selenium nanoparticles to zebrafish (*Danio rerio*) embryogenesis. Nanotoxicology 2017;11:87–97. 28008795 10.1080/17435390.2016.1275866

[47] Asri-Rezaei S , Nourian A, Shalizar-Jalali A, Najafi G, Nazarizadeh A, Koohestani M, Karimi A. Selenium supplementation in the form of selenium nanoparticles and selenite sodium improves mature male mice reproductive performances. Iran J Basic Med Sci 2018;21:577–85. 29942447 10.22038/IJBMS.2018.26023.6397 PMC6015244

[48] Xu C , Qiao L, Guo Y, Ma L, Cheng Y. Preparation, characteristics and antioxidant activity of polysaccharides and proteins-capped selenium nanoparticles synthesized by *Lactobacillus casei* ATCC 393. Carbohydr Polym 2018;195:576–85. 29805014 10.1016/j.carbpol.2018.04.110

[49] Zheng Y , Mao YR, Yuan TF, Xu DS, Cheng LM. Multimodal treatment for spinal cord injury: a sword of neuroregeneration upon neuromodulation. Neural Regen Res 2020;15:1437–50. 31997803 10.4103/1673-5374.274332 PMC7059565

[50] Bhattacharjee A , Basu A, Biswas J, Bhattacharya S. Nano-Se attenuates cyclophosphamide-induced pulmonary injury through modulation of oxidative stress and DNA damage in Swiss albino mice. Mol Cell Biochem 2015;405:243–56. 25920447 10.1007/s11010-015-2415-1

[51] Rao S , Lin Y, Du Y, He L, Huang G, Chen B, Chen T. Designing multifunctionalized selenium nanoparticles to reverse oxidative stress-induced spinal cord injury by attenuating ROS overproduction and mitochondria dysfunction. J Mater Chem B 2019;7:2648–56. 32254998 10.1039/c8tb02520g

[52] Wang HF , Liu XK, Li R, Zhang P, Chu Z, Wang CL, Liu HR, Qi J, Lv GY, Wang GY, Liu B, Li Y, Wang YY. Effect of glial cells on remyelination after spinal cord injury. Neural Regen Res 2017;12:1724–32. 29171439 10.4103/1673-5374.217354 PMC5696855

[53] Kong X , Gao J. Macrophage polarization: a key event in the secondary phase of acute spinal cord injury. J Cell Mol Med 2017;21:941–54. 27957787 10.1111/jcmm.13034 PMC5387136

[54] Kotter MR , Setzu A, Sim FJ, Van Rooijen N, Franklin RJ. Macrophage depletion impairs oligodendrocyte remyelination following lysolecithin-induced demyelination. Glia 2001;35:204–12. 11494411 10.1002/glia.1085

[55] Huang H , Young W, Skaper S, Chen L, Moviglia G, Saberi H, Al-Zoubi Z, Sharma HS, Muresanu D, Sharma A, El MW, Feng S; International Association of Neurorestoratology and The Chinese Association of Neurorestoratology. Clinical neurorestorative therapeutic guidelines for spinal cord injury (IANR/CANR version 2019). J Orthop Translat 2020;20:14–24. 31908929 10.1016/j.jot.2019.10.006 PMC6939117

[56] Xie W , Yang SY, Zhang Q, Zhou Y, Wang Y, Liu R, Wang W, Shi J, Ning B, Jia T. Knockdown of microRNA-21 promotes neurological recovery after acute spinal cord injury. Neurochem Res 2018;43:1641–9. 29934690 10.1007/s11064-018-2580-1

[57] Yin W , Li X, Zhao Y, Tan J, Wu S, Cao Y, Li J, Zhu H, Liu W, Tang G, Meng L, Wang L, Zhu B, Wang G, Zhong M, Liu X, Xie D, Chen B, Ren C, Xiao Z, Jiang X, Dai J. Taxol-modified collagen scaffold implantation promotes functional recovery after long-distance spinal cord complete transection in canines. Biomater Sci 2018;6:1099–108. 29528079 10.1039/c8bm00125a

